# Prognostic and predictive value of TOPK stratified by KRAS and BRAF gene alterations in sporadic, hereditary and metastatic colorectal cancer patients

**DOI:** 10.1038/sj.bjc.6605452

**Published:** 2009-11-24

**Authors:** I Zlobec, F Molinari, M Kovac, M P Bihl, H J Altermatt, J Diebold, H Frick, M Germer, M Horcic, M Montani, G Singer, H Yurtsever, A Zettl, L Terracciano, L Mazzucchelli, P Saletti, M Frattini, K Heinimann, A Lugli

**Affiliations:** 1 Institute for Pathology, University Hospital of Basel, Basel, Switzerland; 2 Institute of Pathology, Locarno, Switzerland; 3 Research Group Human Genetics, Department of Biomedicine, University of Basel, Basel, Switzerland; 4 Pathologie Länggasse, Bern, Switzerland; 5 Institute for Pathology, Cantonal Hospital of Lucerne, Lucerne, Switzerland; 6 Institute for Pathology, Cantonal Hospital of Chur, Chur, Switzerland; 7 Institute for Pathology, Cantonal Hospital of Winterthur, Winterthur, Switzerland; 8 Institute for Histological and Cytological Diagnostics, Aarau, Switzerland; 9 Department of Pathology, University Hospital Zurich, Zurich, Switzerland; 10 Institute for Pathology, Cantonal Hospital of Baden, Baden, Switzerland; 11 Institute for Pathology, Cantonal Hospital of Aarau, Aarau, Switzerland; 12 Institute of Clinical Pathology, Basel, Switzerland; 13 Oncology Institute of Southern Switzerland, Ospedale San Giovanni, Bellinzona, Switzerland

**Keywords:** colorectal cancer, TOPK, *KRAS*, *BRAF*, anti-EGFR therapy

## Abstract

**Background:**

Our aim was to investigate the prognostic and predictive value of the oncogenic MAPKK-like protein T-cell-originated protein kinase (TOPK) stratified by *KRAS* and *BRAF* mutations in patients with sporadic, hereditary and metastatic colorectal cancer (CRC) treated with anti-EGFR therapy.

**Methods:**

Immunohistochemistry (IHC) for TOPK was performed on four study groups. Group 1 included two subgroups of 543 and 501 sporadic CRC patients used to test the reliability of TOPK expression by IHC. In Group 2, representing an additional 222 sporadic CRCs, the prognostic effect of TOPK stratified by KRAS and BRAF was assessed. The prognostic effect of TOPK was further analysed in Group 3, representing 71 hereditary Lynch syndrome-associated CRC patients. In Group 4, the predictive and prognostic value of TOPK was analysed on 45 metastatic patients treated with cetuximab or panitumumab stratified by *KRAS* and *BRAF* gene status.

**Results:**

In both sporadic CRC subgroups (Group 1), associations of diffuse TOPK expression with clinicopathological features were reproducible. Molecular analysis of sporadic CRCs in Group 2 showed that diffuse TOPK expression was associated with KRAS and BRAF mutations (p<0.001) and with poor outcome in patients with either mutation in univariate and multivariate analysis (*P*=0.017). In hereditary patients (Group 3), diffuse TOPK was linked to advanced pT stage. In metastatic patients treated with anti-EGFR therapy (Group 4), diffuse TOPK expression was linked to dismal outcome despite objective response to treatment (*P*=0.01).

**Conclusions:**

TOPK expression is an unfavourable prognostic indicator in sporadic patients with *KRAS* or *BRAF* mutations and also in patients with metastatic disease experiencing a response to anti-EGFR therapies. The inhibition of TOPK, which could benefit 30–40% of CRC patients, may represent a new avenue of investigation for targeted therapy.

The pathogenesis, progression and oncogenic behaviour of colorectal cancer (CRC) are to a large extent regulated by the ERK/MAPK signalling cascade, which activates transcription factors critical for angiogenesis, proliferation, apoptosis, differentiation and metastasis (Fang and Richardson, 2005). In CRC, 30–40% of cases have mutations in the *KRAS* proto-oncogene (Bos *et al*, 1987). Often linked to tumours arising from the chromosomal instability pathway, representing 80–85% of CRC cases, *KRAS* mutations have been associated with increased activity of ERK signalling, thereby promoting transcription of *Elk-1* and *c-Myc* (Bos *et al*, 1987; Jass, 2007). Although evidence with regard to the effect of *KRAS* gene status on prognosis is heavily debated, the majority of published studies suggest a poorer outcome in patients with *KRAS* mutations (Siena *et al*, 2009). Interestingly, patients with Lynch syndrome-associated CRC, representing only 2–3% of all CRC patients, are also found to have a higher frequency of *KRAS* mutation, yet they generally show a favourable clinical outcome (Oliveira *et al*, 2007; Green *et al*, 2009). Downstream of *KRAS* in ERK/MAPK signalling lies *BRAF*, a gene that in sporadic disease is mutated in ∼10% of CRC and is more highly associated with tumours showing microsatellite instability (MSI) (Bos *et al*, 1987; Jass, 2007). Few reports have investigated the prognostic effect of *BRAF* in CRC; however, evidence points to a worse prognosis in patients with mutations in this gene (Samowitz *et al*, 2005; French *et al*, 2008; Ogino *et al*, 2009).

Current regimens for patients with metastatic CRC include anti-EGFR monoclonal antibodies such as cetuximab and panitumumab, both functioning to block the binding of ligands to EGFR, thereby downregulating ERK/MAPK and PI3K/PTEN/AKT pathway signalling (Amado *et al*, 2008; Giusti *et al*, 2008; Segal and Saltz, 2009). New evidence suggests that patients with *KRAS*, *BRAF* or *PTEN* mutations experience fewer clinical responses to these drugs, compared with patients with wild-type tumours; moreover, molecular analysis, particularly of *KRAS*, is routinely being performed in standard molecular pathology laboratories (Lievre *et al*, 2006; Amado *et al*, 2008; Di Nicolantonio *et al*, 2008; Karapetis *et al*, 2008; Au *et al*, 2009; Jimeno *et al*, 2009; Sartore-Bianchi *et al*, 2009). As 30–40% of patients with CRC exhibit mutations in one of these genes in a mutually exclusive manner, their potential for receiving such targeted agents is substantially decreased (Jass, 2007; Di Nicolantonio *et al*, 2008).

Taken together, the identification of novel prognostic and predictive factors, which consider the heterogeneous molecular background of CRC, particularly with regard to *KRAS* and *BRAF* gene status, is warranted. In 2000, a new member of the ERK/MAPK pathway, T-cell-originated protein kinase (TOPK), also known as PDZ-binding kinase, was identified (Abe *et al*, 2000; Gaudet *et al*, 2000). T-cell-originated protein kinase was described as a MAPKK-like protein involved in p38MAPK and JNK signalling, possibly in a cell-type-dependent manner, and was more recently found to be involved in the ERK/MAPK pathway (Matsumoto *et al*, 2004; Nandi *et al*, 2004; Ayllon and O’Connor, 2007; Oh *et al*, 2007). T-cell-originated protein kinase is overexpressed in highly proliferating normal tissues, foetal tissues and in a wide variety of tumours *in vitro*, whereas the inhibition of TOPK is shown to lead to apoptosis in breast and melanoma cell lines (Simons-Evelyn *et al*, 2001; Zhao *et al*, 2001; Matsumoto *et al*, 2004; Nandi *et al*, 2004; Dougherty *et al*, 2005; Park *et al*, 2006; Zykova *et al*, 2006). Most recently, Herrero-Martin *et al* (2009) evaluated TOPK expression in Ewing sarcoma cell lines and found that the inhibition of TOPK led to a decrease in the proliferation rate and an important change in cell growth, indicating that TOPK could have a significant role in Ewing sarcoma biology. Zhu *et al* (2007) systematically assessed this novel molecule in CRC and confirmed its oncogenic potential *in vitro* and *in vivo*. Importantly, they found that, unlike other MEKs that undergo negative phosphorylation loops between themselves and ERK, TOPK could promote malignant transformation by exerting a positive feedback loop on ERK2 activity. However, the prognostic and predictive effect of TOPK in patients with CRC has to date not been explored.

Given its central involvement in ERK/MAPK signalling, we hypothesised that TOPK overexpression is significantly related to *KRAS* and *BRAF* mutations, thereby implicating this gene in the poorer outcome of patients, both in terms of prognosis and response to anti-EGFR therapies. The aim of our study was, first, to determine using two randomised subgroups (*n*=543 and *n*=501) whether TOPK expression leads to reproducible associations with clinicopathological features by immunohistochemistry (IHC) and, second, to determine according to *KRAS* and *BRAF* gene status the prognostic effect of TOPK on 222 sporadic and 71 Lynch syndrome-associated CRC patients, as well as the prognostic and predictive value of TOPK in 45 metastatic CRC patients treated with anti-EGFR agents, cetuximab and panitumumab.

## Methods

### Patients

#### Sporadic CRC patients (Groups 1 and 2)

A total of 1420 primary pre-operatively untreated, unselected sporadic CRC patients treated at the University Hospital of Basel between 1987 and 1996 were included in this study. Haematoxylin and eosin-stained slides were retrospectively collected from the Institute of Pathology, University Hospital of Basel, the Institute of Clinical Pathology, Basel, Switzerland and from the Institute of Pathology, Stadtspital Triemli, Zürich, Switzerland. Histopathological criteria were reviewed by an experienced gastrointestinal pathologist (LT) and included tumour diameter, pT and pN classification, grade of differentiation, histological subtype, presence of vessel invasion, tumour border configuration (pushing/expanding or infiltrating) and presence of peritumoural lymphocytic inflammation at the invasive tumour front (Jass *et al*, 1986). Clinical data including patient age at diagnosis, tumour location and follow-up, local recurrence, distant metastasis and post-operative therapy were retrieved from the patient records, where available. Censored observations included patients who were alive at the last follow-up, those who died for reasons other than CRC or were lost to follow-up. Median survival time was 76 (95% CI 47–137) months; median follow-up was 60.3 months.

#### Lynch syndrome-associated CRC patients (Group 3)

In all, 94 patients with genetically confirmed Lynch syndrome-associated CRC identified from the Swiss Cancer Registry were included in this study. Histopathological criteria were reviewed and included pT, pN, pM classifications and grade of differentiation. Clinical data including patient age at diagnosis, tumour location and follow-up were retrieved from patient records. Censored observations included patients who were alive at last follow-up, those who died for reasons other than CRC or were lost to follow-up. Follow-up period ranged from 0 to 74 years and median follow-up time was 7.1 years (95% CI 5.4–8.7).

#### Metastatic CRC patients (Group 4)

A total of 46 consecutive patients with histologically confirmed metastatic CRC treated at the Oncology Institute of Southern Switzerland, Bellinzona, Switzerland with cetuximab or panitumumab-based regimens were entered into this study. Cetuximab was administered at a standard loading dose of 400 mg m^−2^ over 2 h, followed by weekly dose of 250 mg m^−2^ over 1 h. Panitumumab (6 mg kg^−1^) was administered intravenously every 2 weeks until progression was allocated in two patients who were refractory to oxaliplatin-based and irinotecan-based regimens. With the exception of two patients who received cetuximab as frontline therapy, the others had failed at least one previous chemotherapy regimen. For those patients who progressed on irinotecan-based regimens, cetuximab was administered in combination with these regimens given at the same dose and schedule. Treatment was continued until progressive disease (PD) or toxicity occurred, according to standard criteria. Clinical response was assessed every 6–8 weeks with radiological examination (computerised tomodensitometry or magnetic resonance imaging). The Response Evaluation Criteria in Solid Tumours (RECIST) were adopted for evaluation, and objective tumour response was classified into complete response, partial response (PR), stable disease (SD) and PD. Follow-up time ranged from 0 to 8 years, with a median of 2.0 years and a median survival time of 2.4 (95% CI 2.0–3.4) years.

### Specimen characteristics

For sporadic CRC patients (Groups 1 and 2), a previously described single-punch tissue microarray was constructed including all 1420 tumours and 57 normal mucosa samples as control (Sauter *et al*, 2003; Zlobec *et al*, 2008). Of the 1420 tumours, paraffin-embedded surgical resection specimens were available for 245 cases, which were retrospectively collected from the archives of the Institute of Pathology, University Hospital Basel, Switzerland for subsequent molecular analysis. Second, a multiple-punch tissue microarray including all 94 patients with Lynch syndrome-associated CRCs was constructed. Briefly, tissue blocks were retrieved from the Research Group Human Genetics, Department of Biomedicine, University of Basel. Haematoxylin and eosin slides were re-evaluated and representative areas from the tumour centre, tumour invasive front and adjacent normal mucosa (if available) were identified using a felt-tip pen. Tissue punches 0.6 mm in diameter were taken from these areas and brought into one recipient paraffin block (3 × 2.5 cm) using a homemade semi-automated tissue arrayer. The final tissue microarray contained 297 tissues, taken from 101 different tissue blocks, and included 135 punches from the tumour centre, 78 from the tumour front and 84 samples of normal tissue. Third, for patients with metastatic disease, the corresponding paraffin-embedded tissue blocks were retrospectively collected and whole-tissue sections were cut at 4 μm.

### Assay methods

#### Immunohistochemistry

Immunohistochemistry was carried out for all tumour specimens from Groups 1 to 4 and for normal mucosa samples using anti-TOPK antibody. Tissue microarrays and whole-tissue sections were dewaxed and rehydrated in dH_2_O. After pressure cooker-mediated antigen retrieval in 0.001 M EDTA (pH 8.0), endogenous peroxidase activity was blocked using 0.5% H_2_O_2_. Sections were incubated with 10% normal goat serum for 20 min. After incubation with primary antibody (PBK/TOPK, rabbit polyclonal, dilution 1 : 50, Cell Signalling, Danvers, MA, USA), sections were incubated with HRP-conjugated secondary antibody (DakoCytomation, Glostrup, Denmark) for 30 min at room temperature, immersed in 3-amino-9-ethylcarbazole plus substrate–chromogen (DakoCytomation) for 30 min and counterstained with haematoxylin. Negative control tissues underwent the same protocol with the primary antibody omitted. Tumour cell immunoreactivity was evaluated by an experienced gastrointestinal pathologist (AL) blinded to clinical end points. Tumour cell staining for TOPK was predominantly observed in the cytoplasm, rather than in the nucleus or membrane. The percentage of positive tumour cells per case was scored. Staining intensity was not considered. The inter-observer variability of TOPK scores was assessed on one tissue microarray slide containing 456 cases by a second independent pathologist (MH) from an external institution and blinded to clinicopathological features.

#### Molecular analyses

For groups 2, 3 and 4, MSI analysis along with KRAS (exon 2, codons 12 and 13) and BRAF (exon 15, codon 600) mutational investigations was performed as detailed previously (Frattini *et al*, 2007; Lugli *et al*, 2009). Microsatellite stable and MSI-low status were defined as instability at 0 and 1 markers, respectively. Microsatellite instability-high was characterised by the presence of instability in ⩾2 markers (Umar *et al*, 2004).

### Study design

**Figure 1:**
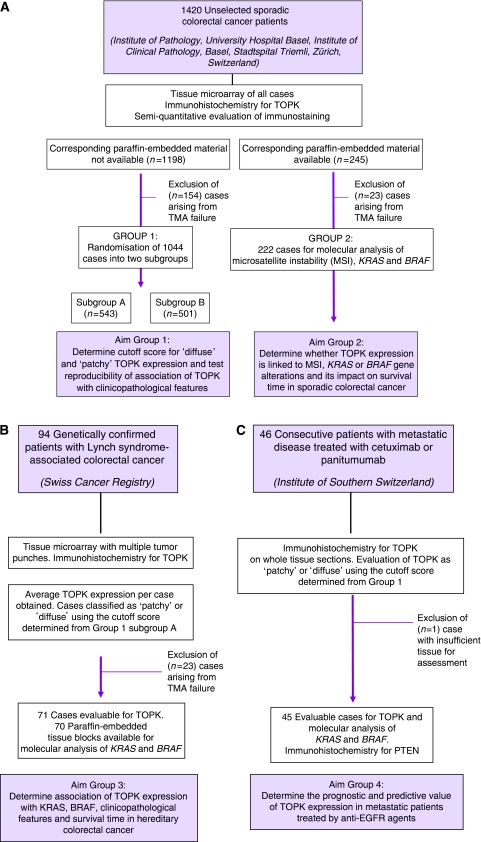
Study design. (**A**) 1420 sporadic colorectal cancers (CRCs) mounted onto tissue microarrays (TMA) underwent immunohistochemistry (IHC) for TOPK and were then subdivided into Group 1 (*n*=1198) and Group 2 (*n*=245) on the basis of the availability of paraffin-embedded material. Excluding 154 cases, Group 1 was randomised into two matched subgroups (*n*=543 and 501), then used to define ‘diffuse’ and ‘patchy’ TOPK expression and to test associations of TOPK with cliniopathological features. In Group 2, 23 cases were excluded. A total of 222 cases with evaluable TOPK IHC were analysed for microsatellite instability (MSI), KRAS and BRAF. The prognostic value of TOPK stratified by KRAS and BRAF gene status was determined. (**B**) TOPK IHC staining was assessable in 71 of 94 Lynch syndrome-associated CRC patients in Group 3. T-cell-originated protein kinase expression was related to KRAS and BRAF mutation, clinicopathological features and cancer-specific survival time. (**C**) TOPK IHC was assessable in 45 of 46 metastatic CRC patients, whereas investigations of MSI, KRAS, BRAF and PTEN were performed. The prognostic and predictive value of TOPK in metastatic CRC patients treated with anti-EGFR agents was evaluated.

The study design is outlined in Figure 1. For study groups 1–3, excluded cases were those resulting from tissue microarray failure, that is, insufficient tissue for evaluation or <50% tumour/punch.

The 1420 sporadic CRCs mounted onto the tissue microarray underwent IHC for TOPK and staining was evaluated semi-quantitatively. These cases were subdivided into two groups on the basis of the availability of corresponding paraffin-embedded material for subsequent DNA extraction (Figure 1A). Group 1 included cases without available tumour blocks (*n*=1198), whereas Group 2 represented cases with available archival paraffin-embedded material (*n*=245).

After exclusion of 154 cases, Group 1 was further randomised into two matched subgroups containing 543 and 501 patients each. The appropriate IHC cutoff score for TOPK for all study groups was determined using subgroup A. Second, the reliability of TOPK expression and its association with clinicopathological features could be determined by analysing both subgroups independently.

After exclusion of 23 cases from Group 2, 222 cases underwent molecular investigations for MSI, KRAS and BRAF. The aim of this study group was to determine the prognostic value of TOPK in CRCs, with subgroup analysis by KRAS and BRAF mutation. Multivariable cancer-specific survival time models were evaluated by including candidate variables such as age, sex, pT and pN classification, vascular invasion and MSI status.

A total of 23 cases were excluded from Group 3 (Figure 1B). The remaining 71 Lynch syndrome-associated CRCs underwent molecular analysis for KRAS and BRAF. The association of TOPK expression with mutational status of KRAS and BRAF, clinicopathological features and cancer-specific survival time, was assessed.

One case of metastatic CRC was excluded from Group 4 because of insufficient material for adequate assessment of TOPK expression (Figure 1C). Immunohistochemistry for PTEN and molecular investigations of MSI, KRAS and BRAF were previously performed (Frattini *et al*, 2007). The prognostic and predictive value of TOPK in this group of patients was analysed, with specific end points of interest being cancer-specific survival time and objective tumour response to anti-EGFR agents.

The use of all patient material was approved by local Ethics Committees.

### Statistical analysis methods

Associations of TOPK with categorical features were investigated by Chi-Square and Fisher's Exact tests where appropriate, and by Student's *t*-test for age. Survival analysis was performed using the Kaplan–Meier method, log-rank test and by multiple Cox regression analysis after verification of the proportional hazards assumption. The appropriate number of variables to be included in regression models was dependent on the frequency of patient deaths in each analysis. We included 1 variable per 10 deaths, to prevent overfitting. Differences in TOPK expression between normal colonic mucosa and tumour were determined using Wilcoxon's rank-sum test for medians. The most clinically relevant cutoff score for TOPK was determined on subgroup A by receiver operating characteristic (ROC) curve analysis for end point survival/death. To prevent overfitting, re-sampling of data was performed by bootstrapping 200 times. The inter-observer variability of TOPK staining was assessed using the intra-class correlation coefficient (ICC), with values of ⩾0.8 indicating excellent agreement. Missing clinicopathological data were assumed to be at random. No imputation was performed; rather, only patients with complete data for all features were included in multivariable analyses. *P*-values <0.05 were considered to be statistically significant.

## Results

### TOPK expression in normal colon versus sporadic CRC

T-cell-originated protein kinase expression in 57 normal colonic mucosa samples was compared with sporadic CRCs from Group 1 (*n*=1044). T-cell-originated protein kinase was highly overexpressed in tumours with a median of 90% positive cell staining compared with 5% positive cell staining in normal tissue (*P*<0.001).

### Inter-observer agreement of TOPK scoring and determination of the cutoff score in CRC

**Figure 2:**
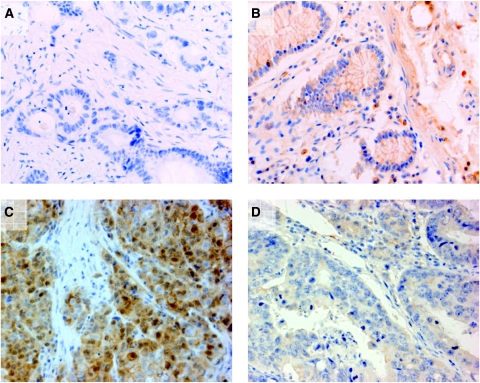
Representative photomicrographs ( × 40) after immunohistochemistry staining with anti-TOPK antibody. (**A**) Colorectal cancer used as a negative control with the primary antibody omitted; (**B**) normal colonic mucosa with negligible cytoplasmic TOPK staining; (**C**) diffuse cytoplasmic TOPK staining in >90% of colorectal tumour cells; and (**D**) patchy cytoplasmic staining of TOPK in ⩽90% of colorectal tumour cells.

Re-evaluation of one tissue microarray slide (*n*=456 CRCs) by a second independent pathologist from an external institution using the same semi-quantitative scoring method resulted in ICC=0.92, indicating excellent agreement. Having established that the evaluation of TOPK staining was reproducible between observers, next, the most appropriate cutoff score to describe tumours as overexpressed for TOPK was evaluated. Using ROC curve analysis, the protein expression value with the highest sensitivity and specificity for patient survival was obtained for subgroup A (Group 1) and was found to be 90% positive for cell staining. This value also coincided with the median expression value of TOPK in sporadic CRCs in Group 1, hence tumours with >90% positive cell staining for TOPK were considered ‘diffuse’, whereas cases with ⩽90% were defined as ‘patchy’ (Figure 2). This definition was subsequently applied to all tumours in this study.

### Group 1: TOPK in sporadic CRC and clinicopathological information

In subgroups A and B, 141 and 111 patients had a diffuse TOPK expression (26 and 28% of cases, respectively). In both randomised subgroups, diffuse TOPK expression was associated with tumour location (more right sided; *P*=0.008 and *P*=0.027) and with high tumour grade (*P*=0.04 and *P*=0.025) (Table 1).

### Group 2: TOPK in sporadic CRC, molecular features and survival time

In Group 2, TOPK was evaluable in 222 cases. Diffuse expression, observed in 63 patients (corresponding to 28% of cases), was linked to tumour location (more right-sided tumours; *P*=0.05), mucinuous histological subtype (*P*=0.027) and poor tumour grade (*P*=0.012) (Table 2).

Mutational investigations gave analysable sequences in 198 cases for *BRAF* and 210 cases for *KRAS* mutations. *BRAF* mutations were observed in 30 cases (15%), whereas *KRAS* mutations occurred in 57 cases (27%). Mutations in *BRAF* (*P*=0.002) and *KRAS* (*P*=0.054) occurred more frequently in patients with diffuse TOPK staining compared with patients with wild-type tumours. As *KRAS* and *BRAF* mutations were mutually exclusive, the relationship of TOPK with either *KRAS* or *BRAF* mutation was evaluated. The diffuse expression found in 36 of 63 (57.1%) patients was significantly associated with mutation in either *KRAS* or *BRAF*, compared with 32.1% of patients with a patchy expression (*P*<0.001).

**Figure 3:**
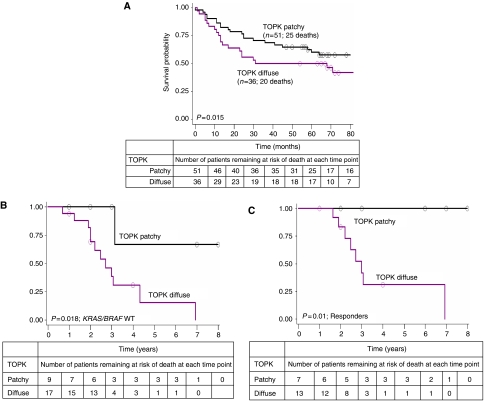
Kaplan–Meier survival curves (**A**) illustrating survival time differences among patients in Group 2 with *KRAS* or *BRAF* mutations stratified by TOPK expression, (**B**) of metastatic colorectal cancer patients illustrating the negative effect of diffuse TOPK expression on prognosis in patients with *KRAS* and *BRAF* wild-type tumours and (**C**) of patients with stable disease or response to anti-EGFR therapy. Tables describe the number of patients at risk of death (alive) at each time point, beginning at the initial time of diagnosis when all patients are alive.

Among patients with *KRAS* or *BRAF* mutations, those with diffuse TOPK expression had a significantly worse prognosis compared with patients with a patchy expression (*P*=0.015) (Figure 3A). The relative risk of death for patients with *KRAS* or *BRAF* mutations was 2.22 (95% CI 1.1–4.4) compared with those showing no mutation in either gene. In multivariate survival analysis with age, pT classification and pN classification, TOPK expression maintained a significant adverse effect on outcome (*P*=0.017; HR=2.42 (95% CI 1.2–5.0)), as well as after adjusting for the prognostic effects of pT classification, pN classification and MSI status (*P*=0.018; HR=2.39 (95% CI 1.2–4.9)) (Table 3).

### Group 3: TOPK in hereditary Lynch syndrome-associated CRC

T-cell-originated protein kinase expression could be assessed in 71 patients with Lynch syndrome-associated CRC. Of the 30 patients with a diffuse TOPK expression (41% of cases), 27 (93.1%) had pT3 or pT4 tumours compared with 68.2% of patients with a patchy expression (*P*=0.014). *KRAS* mutations were found in 22 (31%) patients, whereas mutation in *BRAF* was noted in only one case of genetically confirmed Lynch syndrome. No association of TOPK was observed with either prognosis or *KRAS* mutation status (Table 4).

### Group 4: TOPK in metastatic CRC patients treated with anti-EGFR therapy

Of the 45 metastatic patients treated with cetuximab or panitumumab with evaluable TOPK staining, a wild-type KRAS and BRAF gene status was detected in 32 (71.1%) and 41 (91%) cases, respectively. Diffuse TOPK expression was observed in 19 (82.6%) KRAS wild-type and 21 (91.3%) BRAF wild-type tumours. A highly unfavourable outcome in patients with *KRAS* and *BRAF* wild-type tumours with overexpression of TOPK was noted (*P*=0.018) (Figure 3B). No difference in TOPK staining was found between PTEN loss and overexpression, and the prognostic effect of diffuse TOPK staining in KRAS and BRAF wild-type patients was maintained after adjusting for PTEN status (*P*=0.041). In total, 23 patients (51.1%) had PD, 11 (24.4%) had PR and 11 (24.4%) had SD, with diffuse expression of TOPK occurring in 10 (43.5%), 7 (30.4%) and 6 (26.1%) patients, respectively. Patients having SD or PR to anti-EGFR therapy but with diffuse TOPK expression suffered from poor outcome; in contrast, those with no overexpression of TOPK were alive or censored at 5-year follow-up (*P*=0.01) (Figure 3C). T-cell-originated protein kinase expression was not of predictive value for response to anti-EGFR therapy, either in the entire cohort of patients or when stratified by *KRAS* and *BRAF* mutation status (Table 5).

## Discussion

We report the association of diffuse TOPK expression with specific sporadic CRC features, namely, with right-sided tumour location and higher tumour grade in two large multicentric cohorts of patients and excellent inter-observer reproducibility of TOPK scores. Second, our findings point to the diffuse expression of TOPK as an adverse prognostic factor in patients with sporadic CRC with a *KRAS* or *BRAF* mutation and in metastatic patients with SD or PR after treatment with anti-EGFR-based regimens.

In sporadic CRC, diffuse TOPK expression was associated with the presence of *KRAS* or *BRAF* mutation, underlining the involvement of TOPK in ERK/MAPK signalling. In patients with either *KRAS* or *BRAF* mutations, diffuse expression of TOPK had an adverse effect on 5-year survival. In addition, this unfavourable effect of TOPK expression on outcome was maintained in multivariate analysis, suggesting that TOPK could represent an important prognostic factor in patients with *KRAS-*mutated or *BRAF-*mutated tumours (Andreyev *et al*, 1998; Samowitz *et al*, 2005; French *et al*, 2008; Ogino *et al*, 2009). Although *KRAS* mutations are frequently found in patients with Lynch syndrome-associated CRC despite their favourable prognosis, in this study, no association between TOPK expression and *KRAS* mutation was observed (Oliveira *et al*, 2007). The propensity for more right-sided, poorly differentiated cancers and poorer outcome in patients with *KRAS* or *BRAF* mutation was not found here, despite an association with a more advanced pT stage with diffuse TOPK staining. These results seem to indicate that involvement of TOPK in CRC may be limited to tumours of sporadic origin.

We report that in 45 metastatic CRC patients treated with anti-EGFR agents and with wild-type *KRAS* and *BRAF* gene status, those expressing diffuse TOPK staining suffer from a significant adverse prognosis. In addition, TOPK expression seemed to be unmodified by PTEN status and maintained its adverse effect on outcome in KRAS or BRAF wild-type patients independently of the expression of this molecule. Furthermore, among patients with SD or those with objective response, a diffuse expression of TOPK may act as a highly unfavourable prognostic factor. Together, these results indicate that the activation of MAPK signalling is still possible at the level of TOPK, even in the context of wild-type *KRAS* and *BRAF*, and is unlikely because of loss of PTEN. Therefore, TOPK may act as a prognostic, rather than as a predictive, factor, suggesting that it may be important to consider its expression in metastatic CRC patients with a proficient molecular profile for positive response to anti-EGFR drugs.

Our results suggest that inhibition of TOPK could be beneficial for at least two groups of CRC patients together representing 30–40% of all cases, namely, those with a *KRAS* or *BRAF* mutation and those with metastatic disease supported by several factors. T-cell-originated protein kinase is barely detectable in most normal adult tissues including normal colonic mucosa, whereas it is highly overexpressed in CRC (Zhu *et al*, 2007). Its detection by IHC leads to reproducible associations with clinicopathological features and its evaluation leads to excellent inter-observer agreement. As a MAPKK-like protein, it is a downstream molecule of *KRAS* and *BRAF*, both of which are associated with diffuse expression of TOPK (Roberts and Der, 2007). T-cell-originated protein kinase may itself be an effector of *BRAF*, as phosphorylation of TOPK by RAF has previously been shown (Yuryev and Wennogle, 2003). Therefore, inhibition of TOPK at this level of signalling may have a more significant impact on downregulating deregulated ERK/MAPK signalling. Current MEK inhibitors have led to moderate results (Roberts and Der, 2007). Although blocking MEK1 should lead to a decrease in the phosphorylation of ERK1/2, this process is hindered by a negative feedback loop of ERK1/2 onto MEK1, making inhibition of this molecule to some extent counter productive (Ramos, 2008). T-cell-originated protein kinase, in contrast, has been described as an oncogenic MEK involved in a positive phosphorylation loop with ERK2 (Zhu *et al*, 2007). Therefore, inhibition of TOPK should be expected to successfully decrease the activation of ERK2 and thus its downstream transcription factors. Moreover, TOPK expression in this study seems to be independent of PTEN status. Considering recent evidence suggesting that *PTEN* mutation results in resistance to EGFR-targeted therapies (Sartore-Bianchi *et al*, 2009), the inhibition of TOPK in KRAS and BRAF wild-type patients could represent an approach to improve clinical outcome in patients with either *PTEN* wild-type or mutated cancers.

A limitation of this study is that information on cancer treatment was limited. Subgroup analysis produced results using relatively small sample sizes; therefore, these findings necessitate validation on larger patient cohorts. Nonetheless, our study gives valuable results for several reasons. Four groups of patients were included, representing sporadic, hereditary and metastatic CRC. Patients were treated in different centres and considerable corresponding clinicopathological data and follow-up could be obtained. Whole-tissue sections and two tissue microarrays were evaluated, the largest containing more than 1000 tumours, the second with multiple tissue punches taken from the same patient representing different tumour areas. Finally, the cohort of metastatic CRC was well characterised with respect to both clinicopathological treatment and molecular features.

Although several study groups have investigated the functional role of TOPK in different tumour types, this seems to be the first assessment of the prognostic and predictive value of this protein in CRC. In conclusion, TOPK seems to be a valuable prognostic factor in patients with sporadic CRC with *KRAS* or *BRAF* gene mutations, as well as in patients with metastatic disease who respond to anti-EGFR therapies. If confirmed prospectively, the inhibition of TOPK may represent a novel avenue of investigation for targeted treatment in patients with CRC, especially for the early identification of patients with a worse prognosis, although experiencing disease control after anti-EGFR drug administration.

## Figures and Tables

**Table 1:** Group 1: immunohistochemical expression of TOPK (patchy or diffuse) and association with clinicopathological features in both randomized subgroups A and B

	**Subgroup A**			**Subgroup B**		
**Clinicopathological features**	**Patchy *N* (%)**	**Diffuse *N* (%)**	***P*-value**	**Patchy *N* (%)**	**Diffuse *N* (%)**	***P*-value**
*Gender*						
Female	211 (52.5)	75 (53.2)	0.886	200 (51.3)	62 (55.9)	0.395
Male	191 (47.5)	66 (46.8)		190 (48.7)	49 (44.1)	
						
*Tumour location*						
Left sided	136 (34.2)	36 (25.7)	0.008	132 (34.3)	33 (30.2)	0.027
Right sided	131 (32.9)	64 (45.7)		119 (30.9)	46 (42.2)	
Rectum	131 (32.9)	40 (28.6)		134 (34.8)	30 (27.5)	
						
*Histological subtype*						
Mucinous	30 (7.5)	16 (11.4)	0.154	26 (6.7)	11 (9.9)	0.249
Non-mucinous	372 (92.5)	125 (88.7)		364 (93.3)	100 (90.1)	
						
*pT stage*						
pT1–2	77 (19.4)	23 (16.7)	0.471	67 (17.5)	18 (16.4)	0.782
pT3–4	319 (80.6)	115 (83.3)		316 (82.5)	92 (83.6)	
						
*pN stage*						
pN0	194 (49.6)	71 (52.2)	0.603	198 (52.4)	55 (51.9)	0.928
pN1–2	197 (50.4)	65 (47.8)		180 (47.6)	51 (48.1)	
						
*pM stage*						
pM0	116 (81.7)	53 (80.3)	0.812	124 (81.1)	42 (82.4)	0.836
pM1	26 (18.3)	13 (19.7)		29 (18.9)	9 (17.7)	
						
*Tumour grade*						
G1–2	346 (87.4)	112 (80.6)	0.04	341 (89.5)	88 (81.5)	0.025
G3	50 (12.6)	27 (19.4)		40 (10.5)	20 (18.5)	
						
*Vascular invasion*						
Absence	274 (69.4)	110 (79.1)	0.028	285 (74.8)	79 (72.5)	0.624
Presence	121 (30.6)	29 (20.9)		96 (25.2)	30 (27.5)	
						
*Local recurrence*						
Absence	73 (52.9)	43 (66.2)	0.075	90 (59.6)	30 (58.8)	0.922
Presence	65 (47.1)	22 (33.9)		61 (40.4)	21 (41.2)	
						
*Post-operative therapy*						
No	104 (7.3)	53 (80.3)	0.344	129 (84.9)	37 (72.6)	0.048
Yes	36 (25.7)	13 (19.7)		23 (15.1)	14 (27.5)	
						
	**Mean (min, max)**			**Mean (min, max)**		
Age (years)	69.9, 36–96	70.1, 39–93	0.795	70.1, 30–96	70.7, 46–96	0.654
						
	**Rate (95% CI)**			**Rate (95% CI)**		
*Survival time*						
5-year	55.7 (50–61)	62.9 (53–71)	0.337	58.6 (53–64)	54.9 (44–65)	0.843

Abbreviations: CI=confidence interval; *N*=frequency; TOPK=T-cell-originated protein kinase.

**Table 2:** Group 2: immunohistochemical expression of TOPK (patchy or diffuse) and association with clinicopathological and molecular features in sporadic colorectal cancer

	**Group 2 (*N* (%))**		
**Clinicopathological features**	**Patchy**	**Diffuse**	***P*-value**
*Gender*			
Female	87 (54.7)	28 (44.4)	0.167
Male	72 (45.3)	35 (55.6)	
			
*Tumour location*			
Left sided	42 (26.4)	18 (28.6)	0.05
Right sided	47 (29.6)	27 (42.9)	
Rectum	70 (44.0)	18 (28.6)	
			
*Histological subtype*			
Mucinous	6 (3.8)	8 (12.7)	0.027
Non-mucinous	153 (96.2)	55 (87.3)	
			
*pT stage*			
pT1–2	36 (22.8)	13 (21.3)	0.815
pT3–4	122 (77.2)	48 (78.7)	
			
*pN stage*			
pN0	84 (54.6)	36 (59.0)	0.552
pN1–2	70 (45.5)	25 (41.0)	
			
*Tumour grade*			
G1–2	154 (97.5)	54 (88.5)	0.012
G3	4 (2.5)	7 (11.5)	
			
*Vascular invasion*			
Absence	112 (70.9)	43 (70.5)	0.954
Presence	46 (29.1)	18 (29.5)	
			
*KRAS*			
Wild type	117 (76.5)	36 (63.2)	0.054
Mutation	36 (23.5)	21 (36.8)	
			
*BRAF*			
Wild type	129 (89.6)	39 (72.2)	0.002
Mutation	15 (10.4)	15 (27.8)	
			
*KRAS*/*BRAF*			
Both wild type	108 (67.9)	27 (42.9)	< 0.001
*KRAS* or *BRAF* mutation	51 (32.1)	36 (57.1)	
			
*Microsatellite status*			
Stable/low	126 (79.3)	48 (76.2)	0.618
High	33 (20.8)	15 (23.8)	
			
	**Mean (min, max)**		
*Age (years)*			
Mean, range	67.6, 43–95	69.7, 44–89	0.156
			
	**Rate (95% CI)**		
*5-year survival time*			
All patients	54.6 (47–63)	52.3 (39–64)	0.719

Abbreviations: CI=confidence interval; *N*=frequency; TOPK=T-cell-originated protein kinase.

**Table 3:** Two multivariable analyses of TOPK expression in sporadic *KRAS*-mutated or *BRAF*-mutated colorectal cancer patients

	**Analysis 1**			**Analysis 2**	
**Variable**	**Hazard ratio (95% CI)**	***P*-value**	**Variable**	**Hazard ratio (95% CI)**	***P*-value**
*TOPK expression*			*TOPK expression*		
Patchy	1.0	0.017	Patchy	1.0	0.018
Diffuse	2.42 (1.2–5.0)		Diffuse	2.39 (1.2–4.9)	
					
*Age*			*pT stage*		
Baseline year	1.0	0.004	pT1–2	1.0	0.826
	1.06 (1.1–1.1)		pT3–4	1.13 (0.4–3.3)	
					
*pT stage*			*pN stage*		
PT1–2	1.0	0.542	pN0	1.0	0.177
PT3–4	0.71 (0.3–2.1)		pN1–2	1.63 (0.8–3.3)	
					
*pN stage*			*MSI status*		
pN0	1.0	0.352	MSS/MSI-L	1.0	0.112
pN1–2	1.38 (0.7–2.7)		MSI-H	1.9 (0.9–4.2)	

Abbreviations: CI=confidence interval; MSI=microsatellite instability; MSI-L=MSI low; MSI-H=MSI high; MSS=microsatellite stable; TOPK=T-cell-originated protein kinase.

**Table 4:** Group 3: immunohistochemical expression of TOPK (patchy or diffuse) and association with clinicopathological and molecular features in hereditary Lynch syndrome-associated colorectal cancers

	**Group 3 (*N* (%))**		
**Clinicopathological features**	**Patchy**	**Diffuse**	***P*-value**
*Gender*			
Female	22 (53.7)	18 (60.0)	0.635
Male	19 (46.3)	12 (40.0)	
			
*Tumour location*			
Left sided	18 (46.2)	11 (39.3)	0.21
Right sided	12 (30.7)	14 (50.0)	
Rectum	9 (23.1)	3 (10.7)	
			
*pT stage*			
pT1–2	12 (31.6)	2 (6.9)	0.014
pT3–4	26 (68.4)	27 (93.1)	
			
*pN stage*			
pN0	23 (65.7)	14 (51.9)	0.27
pN1–2	12 (34.3)	13 (48.2)	
			
*pM stage*			
pM0	14 (70.0)	5 (55.6)	0.675
pM1	6 (30.0)	4 (44.4)	
			
*Tumour grade*			
G1–2	24 (72.7)	18 (64.3)	0.478
G3	9 (27.3)	10 (35.7)	
			
*KRAS*			
Wild type	28 (68.3)	20 (61.0)	1.0
Mutation	13 (31.7)	9 (31.0)	
			
*BRAF*			
Wild type	38 (100.0)	28 (96.6)	0.433
Mutation	0 (0.0)	1 (3.5)	
			
*KRAS*/*BRAF*			
Both wild type	28 (68.3)	19 (65.5)	1.0
*KRAS* or *BRAF* mutation	13 (31.7)	10 (34.5)	
			
*Microsatellite status*			
Stable/low			
High	41 (57.3)	30 (42.3)	0.192
			
	**Mean (min, max)**		
*Age (years)*			
Mean, range	45.3, 24–73	47.4, 27–83	0.492
			
	**Rate (95% CI)**		
*5-year survival time*			
All patients	87.5 (73–95)	88.7 (69–96)	0.66

Abbreviations: CI=confidence interval; EGFR=epidermal growth factor receptor; *N*=frequency; TOPK=T-cell-originated protein kinase.

**Table 5:** Group 4: Immunohistochemical expression of TOPK (patchy or diffuse) and clinicopathological and molecular features in metastatic colorectal cancer patients treated with anti-EGFR therapy

	***N* (%)**		
**Clinicopathological features**	**Patchy**	**Diffuse**	***P*-value**
*Age (years)*			
Mean, range	65.7, 48–82	60.7, 26–79	0.113
			
*Gender*			
Female	8 (36.4)	9 (39.1)	0.848
Male	14 (63.6)	14 (60.9)	
			
*Clinical response*			
Progressive disease	13 (59.1)	10 (43.5)	0.528
Partial response	4 (18.2)	7 (30.4)	
Stable disease	5 (22.7)	6 (26.1)	
			
*KRAS codon 12 and 13*			
Wild type	13 (59.1)	19 (82.6)	0.082
Mutation	9 (40.9)	4 (17.4)	
			
*BRAF codon 600*			
Wild type	20 (90.9)	21 (91.3)	1.0
Mutation	2 (9.1)	2 (8.7)	
			
*KRAS*/*BRAF*			
Both wild type	11 (50.0)	17 (73.9)	0.098
*KRAS* or *BRAF* mutation	11 (50.0)	6 (26.1)	
			
*Microsatellite status*			
Stable/low	22 (100.0)	22 (100.0)	
High			
			
*EGFR amplification*			
No copy number gain	4 (19.1)	3 (13.0)	0.693
Copy number gain	17 (81.0)	20 (87.0)	
			
*PI3KCA*			
Loss	19 (86.4)	20 (87.0)	1.0
Overexpression	3 (13.6)	3 (13.0)	
			
*PTEN*			
Loss	11 (50.0)	6 (26.1)	0.09
Overexpression	11 (50.0)	17 (73.9)	
			
	**Rate (95% CI)**		
*5-year survival time*			
All patients	34.5 (11–60)	13.5 (1–40)	0.473
Either *KRAS* or *BRAF* mutation	18.2 (3–44)	16.7 (0–51)	0.887
Both wild type *KRAS* and *BRAF*	66.7 (5–95)	15.3 (1–45)	0.018
Stable disease or response	100	31.3 (8–59)	0.01

Abbreviations: CI=confidence interval; EGFR=epidermal growth factor receptor; *N*=frequency; TOPK=T-cell-originated protein kinase.
